# IgA vasculitis (Henoch-Schönlein purpura) nephritis and psoriasis in a child: is there a relationship?

**DOI:** 10.1590/2175-8239-JBN-2020-0101

**Published:** 2021-02-15

**Authors:** Ana Sofia Vaz, Raquel Penteado, Carolina Cordinhã, Carmen Carmo, Clara Gomes

**Affiliations:** 1 Centro Hospitalar e Universitário de Coimbra, Departamento de Pediatria, Serviço de Pediatria Ambulatória, Unidade de Nefrologia Pediátrica, Coimbra, Portugal.

**Keywords:** Psoriasis, Púrpura de Schoenlein-Henoch, Glomerulonephritis, IGA, Psoríase, Púrpura de Henoch-Schönlein, Glomerulonefrite por IGA

## Abstract

**Background:**

Psoriasis is a chronic immune-mediated disorder that primarily affects the skin in both adults and children but can also have systemic involvement, particularly with arthritis and kidney injury. IgA nephropathy is the most frequent kidney disorder associated with psoriasis. Approximately one third of all cases of psoriasis begin in childhood, but association between psoriasis and renal disorders has scarcely been reported in pediatric patients. Henoch-Schönlein purpura (HSP) is a systemic vasculitis characterized by IgA deposits in the vessel walls of affected organs and in the mesangium of the kidney. HSP nephritis histopathology is identical to IgA nephropathy.

**Case report:**

A 6-year-old boy with recent onset of psoriasis developed HSP with kidney involvement, clinically manifested by nephrotic-range proteinuria and hematuria. Kidney biopsy revealed fibrocellular glomerular crescents and mesangial IgA deposits compatible with IgA nephropathy. Treatment with systemic corticosteroids led to the control of hematuria, but as nephrotic-range proteinuria persisted, cyclophosphamide was added, leading to a gradual decrease in proteinuria.

**Conclusions:**

We propose an underlying common mechanism in the pathogenesis of both HSP and psoriasis, involving a dysregulation of the IgA-mediated immune response, which could predispose to both entities as well as to kidney damage and IgA nephropathy in these patients.

## Resumo

**Histórico:**

A psoríase é uma doença crônica imunomediada que afeta principalmente a pele tanto em adultos quanto em crianças, mas também pode ter envolvimento sistêmico, particularmente com artrite e lesão renal. A nefropatia por IgA é o distúrbio renal mais frequentemente associado à psoríase. Aproximadamente um terço de todos os casos de psoríase começam na infância, mas a associação entre psoríase e distúrbios renais tem sido pouco relatada em pacientes pediátricos. A Púrpura de Henoch-Schönlein (PHS) é uma vasculite sistêmica caracterizada por depósitos de IgA nas paredes dos vasos de órgãos afetados e no mesângio do rim. A histopatologia da nefrite da PHS é idêntica à da nefropatia por IgA.

**Relato de caso:**

Um menino de 6 anos de idade com início recente de psoríase desenvolveu PHS com envolvimento renal, clinicamente manifestado por proteinúria nefrótica e hematúria. A biópsia renal revelou crescentes fibrocelulares glomerulares e depósitos mesangiais de IgA compatíveis com a nefropatia por IgA. O tratamento com corticosteróides sistêmicos levou ao controle da hematúria, mas como a proteinúria nefrótica persistiu, a ciclofosfamida foi adicionada, levando a uma diminuição gradual da proteinúria.

**Conclusões:**

Propomos um mecanismo comum subjacente na patogênese tanto da PHS quanto da psoríase, envolvendo uma desregulação da resposta imune mediada por IgA, que poderia predispor a ambas as entidades, bem como a danos renais e nefropatia por IgA nesses pacientes.

## Introduction

Psoriasis is a common chronic immune-mediated inflammatory disorder that in the past was considered to be limited to the skin, but recent evidence strongly suggests far-reaching systemic effects, including kidney damage. The so-called "psoriatic nephropathy" has been particularly associated with certain glomerular diseases including IgA nephropathy (IgAN) as one of the most common. Disease severity, presence of arthritis, and age have been reported as risk factors for chronic kidney disease development in psoriatic patients^1^^-^ 5.

More documented in adults, cases of association between psoriasis and kidney disorders in children are rare^6^^-^ 9.

Henoch-Schönlein purpura (HSP) is the most common systemic vasculitis in childhood, characterized by deposits of IgA-containing immune complexes in the vessel walls of affected organs and in the mesangium of the kidney. Histologically, the appearance of HSP nephritis is identical to IgA nephropathy, and recent studies explored a pathogenesis common to both conditions^10^^,^ 11.

HSP is presently called IgA vasculitis, and the name HSP nephritis has been changed to IgA vasculitis nephritis. As IgA nephropathy, IgA vasculitis, and IgA vasculitis nephritis are frequently used along this paper, the authors opted for the use of the previous designations, in order to simplify reading.

To our knowledge, this is the first published case report of a child with psoriasis who developed renal disease compatible with HSP nephritis.

## Case Report

**Figure 1:**
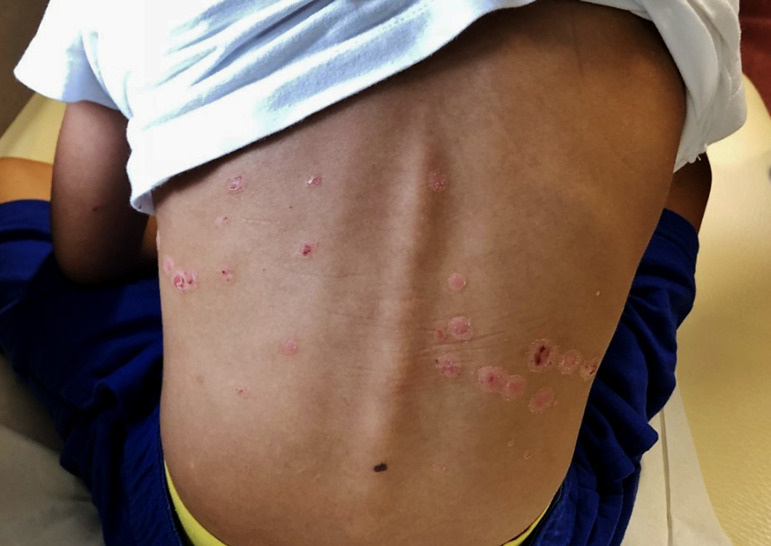
Psoriatic skin lesions in the dorsal region.

**Figure 2:**
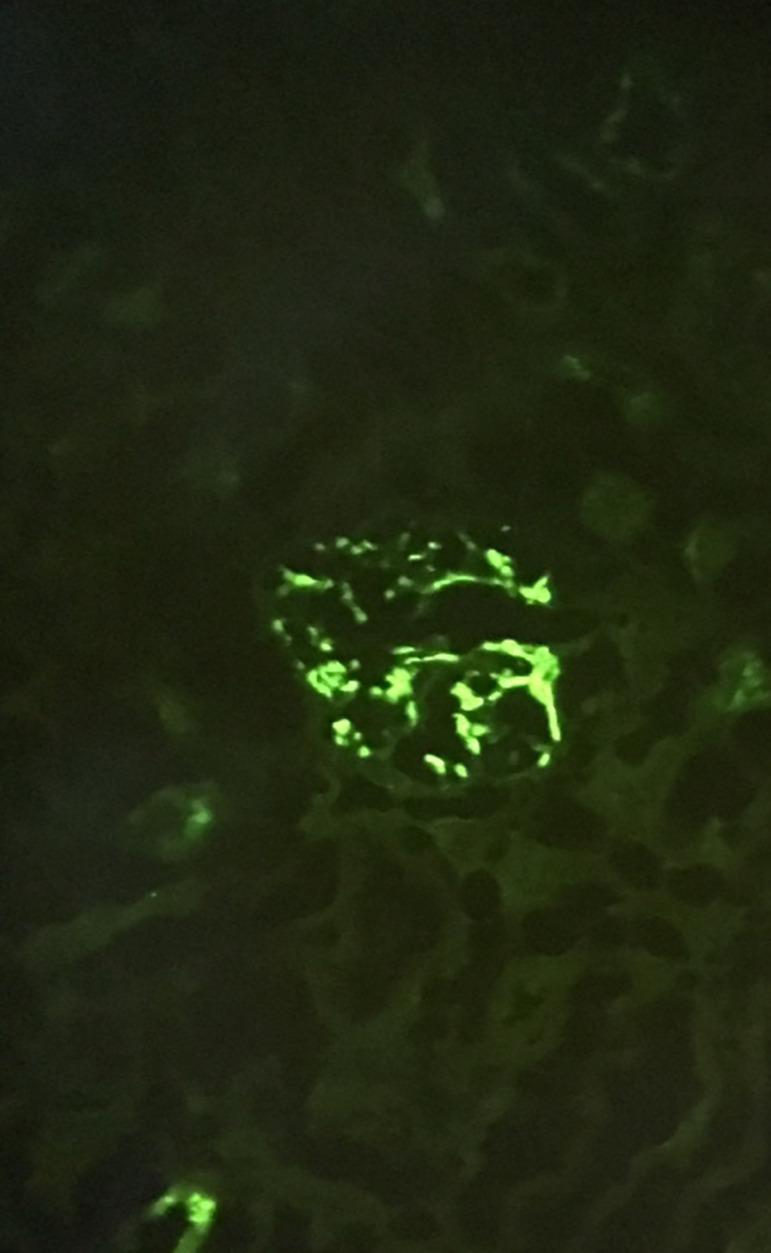
Direct immunofluorescence: mesangial IgA granular deposits.

**Graph 1:**
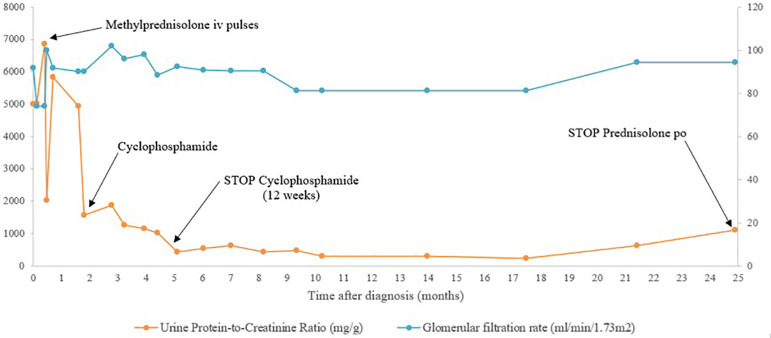
Proteinuria and renal function during treatment.

A 6-year-old boy presented with erythematous scaly lesions on the scalp and back. Clinical diagnosis of psoriasis was established and he started treatment with topical corticosteroid (methylprednisolone ointment). Partial clinical improvement of the lesions was noticed, but residual plaques persisted (Figure 1). Approximately one month later, purpuric lesions appeared in the infrapopliteal region, gluteal area, and upper limbs. He had no fever, no arthralgia, and no gastrointestinal or genitourinary symptoms. A skin biopsy in the affected area was performed and revealed a small-vessel vasculitis affecting the papillary dermis. He continued topical corticosteroid treatment in the newly affected areas with good clinical response. Some days later, he performed a urinalysis that showed microscopic hematuria and nephrotic-range proteinuria (urine protein-to-creatinine ratio (UPCR) of 4998 mg/g). These urinalysis abnormalities persisted in the following two months although he always maintained normal blood pressure, had no edemas or urine macroscopic abnormalities, and had normal serum albumin, creatinine, and lipid profile. A thorough investigation showed no abnormal results, including measurement of circulating complement proteins (C3 and C4), immunoglobulins (IgA), ANA, anti-dsDNA and ENA antibodies, and serologic tests for hepatitis B virus, HIV, and Epstein-Barr virus (EBV). Kidney and bladder ultrasound was normal. Nearly three months after the first altered urinalysis, *de novo* macroscopic hematuria was noticed, concomitant with abdominal pain and vomiting. Nephrotic-range proteinuria (10 mg/m^2^/H) persisted. Kidney biopsy histopathology revealed mesangial proliferation and fibrocellular crescents in 6/16 glomeruli with fibrinoid necrosis, and direct immunofluorescence showed predominant mesangial IgA granular deposits (Figure 2). These aspects were compatible with IgA nephropathy, Haas class III. Three methylprednisolone pulses of 30 mg/kg/day were administered, with subsiding macroscopic hematuria. He maintained oral prednisolone (60 mg/m^2^/day) for 4 weeks. During this treatment period, the psoriatic lesions clinically improved but the nephrotic-range proteinuria persisted (Graph 1). For that reason, cyclophosphamide (2 mg/kg/day) was initiated, concomitantly with progressive withdrawal of prednisolone. Nine weeks later, proteinuria decreased to a non-nephrotic-range (UPCR < 2000 mg/g) but persisted above 1000 mg/g and therefore enalapril was started. He completed 12 weeks of cyclophosphamide. Proteinuria continued to decrease and was negative one month later. Kidney function remained normal. Psoriatic lesions worsened with the progressive tapering of prednisolone dose, in spite of cyclophosphamide. Topical corticosteroid (mometasone) and calcitriol ointment were started but clinical improvement was negligible. He then started phototherapy with significant clinical improvement.

## Discussion

Psoriasis primarily affects the skin in both children and adults, but can have systemic involvement and is associated with several comorbidities including kidney damage. It has been reported that psoriasis, particularly moderate to severe, is associated with an increased risk of incident chronic kidney disease (CKD), independent of traditional risk factors^1^^-^ 3, and severe psoriasis was proposed by some authors as an independent risk factor for end-stage CKD^1^. The same authors found that the relative risk of CKD is especially high among younger patients^1^. Nevertheless, specific kidney diseases observed in psoriasis patients remain poorly defined. IgA nephropathy (IgAN) is the most common glomerulonephritis, presenting with hematuria, proteinuria, and occasionally with reduction of glomerular filtration rate (GFR)^5^.

The exact mechanism of psoriasis leading to kidney injury remains unclear. Chronic inflammation with higher atherosclerotic burden and directly induced kidney inflammation and damage by psoriasis have been suggested^3^. Increased concentration of circulating IgA immune complexes has also been demonstrated as a possible contribution to kidney immune-mediated damage^5^. Systemic treatment of psoriasis with nephrotoxic drugs is another possible cause^5^.

Psoriasis begins in childhood in almost one third of cases and the published incidence rates in children have more than doubled since 1970. However, the epidemiology data available to date are limited^6^. Cases of association between psoriasis and renal disorders in children are very scarce^7^^-^ 9.

HSP, presently called IgA vasculitis (IgAV), is the most common systemic vasculitis in childhood presenting with a tetrad of purpura, arthritis or arthralgia, abdominal pain, and kidney disease. Its characteristic pathological feature is the deposition of IgA-containing immune complexes in the blood vessel walls of affected organs and in the mesangium of the kidney. Kidney involvement is reported in 20-55% of children, and HSP nephritis has its name changed to IgA vasculitis nephritis (IgAVN). The most common finding is microscopic hematuria. Proteinuria of variable degree and/or hypertension might also be present. Kidney function is usually normal but significant kidney impairment can occasionally occur in the case of progressive glomerulonephritis. Every child with HSP should have urinalysis performed at diagnosis and during follow-up^10^. In our patient, urinalysis was not performed at diagnosis but only some days later, which led to a delay in the recognition of renal involvement.

Light microscopy findings in HSP nephritis range from mild mesangial proliferation to severe crescentic glomerulonephritis. Diffuse mesangial IgA deposits seen on immunofluorescence are the hallmark of HSP nephritis, and co-deposition of C3 complement (75%) might also be present^10^.

In our patient, light microscopy revealed inflammatory infiltrate and fibrocellular crescents in some of the glomeruli, and immunofluorescence showed predominance of IgA granular deposits.

Histologically, the appearance of HSP nephritis is identical to IgA nephropathy, and recent studies in patients with HSP and IgA nephropathy have detailed a potential role of IgA_1_ in the pathogenesis of both conditions. Abnormal glycosylation of IgA_1_ molecules predisposes patients with HSP to formation of large immune complexes, whose hepatic clearance is impaired. Consequently, they deposit in small vessels and trigger an immune response leading to an inflammatory reaction presenting as clinical signs and symptoms^10^. However, whether IgAN and HSP nephritis are the same disease is controversial. Li et al. have found some differences in the pathogenesis of both conditions^11^.

Management of HSP includes supportive care and, in some cases, immunosuppressive treatment. There is a European consensus for the diagnosis and management of HSP/IgAV, and HSP nephritis/IgAVN in particular, named SHARE initiative^12^. For patients with mild HSP nephritis, oral prednisolone should be used as first-line treatment. In cases of persistent proteinuria or moderate HSP nephritis, addition of azathioprine (AZA) or mycophenolate mofetil (MMF) may also be used. For severe HSP nephritis, recommendations include high-dose corticosteroids and intravenous cyclophosphamide to induce remission and lower doses of corticosteroids combined with AZA or MMF as maintenance treatment^12^. Plasmapheresis has also been used in children with rapidly progressive glomerulonephritis, but it is difficult to assess its efficacy due to selection bias (it is used in the most severe cases) and concurrent administration of other immunosuppressive treatments. Recent studies in children with HSP nephritis and nephrotic syndrome suggest a potential benefit of cyclosporine A in achieving remission of proteinuria and histological improvement of nephritis in follow-up kidney biopsies^10^^,^ 12. In children with HSP who have kidney involvement with persistent proteinuria, irrespective of whether they are receiving prednisolone or other immunosuppressive treatment, an angiotensin-converting enzyme inhibitor or angiotensin receptor blocker should be considered to prevent and/or limit secondary glomerular injury^12^.

There are currently no international standardized guidelines for medical treatment of pediatric psoriasis. To date, treatment is primarily based on published case reports, guidelines for adult psoriasis, expert opinions, and experience with these drugs in other pediatric disorders. The range of psoriasis treatments has expanded over the past several years, and multiple topical agents (corticosteroids, vitamin D analogs, calcineurin inhibitors, dithranol), phototherapy, and systemic (methotrexate, cyclosporine, retinoids, fumaric acid esters) and biological agents (TNF-a and IL-12/23 blockers) are available^6^.

In our patient, although psoriatic lesions improved with systemic corticosteroid treatment and macroscopic hematuria subsided, nephrotic-range proteinuria persisted, which led to the choice of cyclophosphamide as secondary immunosuppressive treatment. However, with prednisone tapering, psoriasis lesions worsened and topical treatment (calcitriol and topical corticosteroids) was insufficient, pointing to the need for a more effective treatment. Phototherapy was the choice, with a good response. To our knowledge this is the first case report of renal disease and HSP occurring in a child with psoriasis.

As previously mentioned, both psoriasis and HSP have been shown to have IgA abnormalities in their pathogenesis. HSP and HSP nephritis are characterized by IgA immune complex-mediated mesangial cell proliferation. The transferrin receptor (TfR), highly expressed in the mesangium of HSP, acts as an IgA1 receptor, and IgA1-TfR interaction is dependent on glycosylation and multimerization of IgA^13^. Damasiewicz-Bodzek et al. showed increased glycosylation of all peptides including IgA as a result of stimulation of the immune system by oxidative stress in psoriatic patients. This element could be a link between psoriasis and the increased prevalence of atherosclerosis, cardiovascular disease, diabetes mellitus and other diseases, as well as IgAN^5^. We suggest that these common pathways involving IgA1 and IgA1-IgG complexes may be associated with the concomitant emergence of both psoriasis and HSP nephritis in the same patient. Furthermore, we question if chronic inflammation in psoriasis could have a contributory role in nephritis and HSP pathogenesis, as complement pathway activation and inflammatory cells recruitment have been demonstrated in psoriatic IgAN.

## Conclusion

Psoriasis and HSP seem to have a partially common pathogenesis involving IgA immune dysregulation, which might have contributed to IgA nephropathy development in our patient, along with other mechanisms such as psoriasis-associated chronic inflammation. Further studies are needed in order to explore these pathogenetic mechanisms.
